# Hypothermia during Surgical Treatment of Type A Aortic Dissection: A 16 Years' Experience

**DOI:** 10.1155/2020/3893261

**Published:** 2020-01-25

**Authors:** Francesco Macrina, Maria Cristina Acconcia, Luigi Tritapepe, Mizar D'abramo, Wael Saade, Alessandra Capelli, Fabio Miraldi

**Affiliations:** Department of Cardiovascular, Respiratory, Nephrologic, Anesthesiologic, and Geriatric Sciences, UOD Anesthesia and Intensive Care in Cardiac Surgery, Sapienza University of Rome, Rome, Italy

## Abstract

Acute aortic dissection (AAD) is among the most challenging cases for surgical treatment and requires procedural expertise for its safe conduct. Aortic surgery has undergone several changes over the last years, especially concerning cerebral protection. The brilliant results obtained with the aid of selective anterograde cerebral perfusion led to a progressive increase of circulatory arrest temperature, with the rise of safe time along with a reduction of the extracorporeal circulation time and hypothermia-related side effects. However, there is still no definitive consensus concerning the optimal range of temperature to be used during circulatory arrest. *Objectives.* This is a retrospective observational study, and we examined 16-year trends in the presentation, diagnosis, hospital outcome and treatment of A AAD type. In our Cardiac Surgery Unit in Policlinico Umberto I of Rome, our analysis focused on patients, who received ACP during aortic surgery and we analyzed the differences between two distinct groups based on the lowest temperature reached during CPB conduction: Lower Temperature Group (LT) (*T* < 24°C) versus Higher Temperature Group (HT) (*T* ≥ 24°C) arrest circulation temperature. *Methods.* Data from 241 patients enrolled between August 2002 and March 2018 were analyzed. Patients were divided according to the lowest temperature reached into 2 groups: Lower Temperature group (LT) (94 patients) and Higher Temperature Group (HT) (147 patients). *Results.* Our results showed a significant reduction of in-hospital mortality and in-hospital results in patients with higher CPB temperature. The global incidence of complications was statistically reduced in HT group: we found a statistical significant reduction of intestinal ischemia, and a similar trend also for other complications analyzed, such as infections. Since the two groups were similar for type of surgical procedures, we considered these differences depending on the lower temperature value reached, according to the current literature. Conclusions. We found a significantly higher mortality in patients with lower temperature during CPB and a global reduction of complications and in particular a significant reduction of intestinal ischemia in patients with higher temperature during CPB. We found a similar trend in other fields of investigations, so we can conclude that circulatory arrest performed at temperature ≥24°C nasopharyngeal temperature associated with ACP is a safe strategy for aortic surgery for AAD.

## 1. Background

Aortic dissection, is among the most challenging cases for surgical treatment and requires procedural expertise for its safe conduct. Aortic surgery has undergone several changes over the last years, especially concerning cerebral protection. Three main strategies for cerebral protection have been developed and studied. These are deep hypothermic circulatory arrest (DHCA), retrograde cerebral perfusion (RCP) and antegrade cerebral perfusion (ACP).

The recent trend among cardiothoracic surgeons is to avoid DHCA, where hypothermia was the only protection for the whole body, in favor of adjunctive perfusion techniques, RCP and ACP. This led to a different approach regarding circulatory arrest temperature value. According to an expert Consensus [1], hypothermia has been classified into four categories.

### 1.1. Absolute Hypothermia

Absolute hypothermia has been defined as a temperature below or equal to 14°C. At such temperature, between 14% and 22% of pts do not reach electroencephalography (EEG) electric silence. Safe time is estimated to be 30–40 minutes. This temperature range has now been abandoned due to the negative consequences of longer cooling and rewarming time. The use of absolute hypothermia did not provide significant advantages, since the decrease in metabolism is small in relation to deep hypothermia (DH) [2] and in addition to all the problems associated with DH, it should be added that it greatly lengthens the cardiopulmonary bypass (CPB) time, causes a loss of brain self-regulation and promotes immunosuppression.

### 1.2. Deep Hypothermia

Deep hypothermia (DH) has been defined as a temperature between 14.1°C and 20°C. At 20°C, 75–88% of patients do not reach EEG electric silence. Estimated circulatory arrest safe time is 20–30 minutes.

### 1.3. Moderate Hypothermia

Moderate hypothermia (MH) has been defined as a temperature between 20.1°C and 28°C. At 28°C, 99–100% of patients do not reach EEG electric silence.

Circulatory arrest safe time is estimated to be 10–20 minutes.

Cerebral activity rises by 37% between 20°C and 25°C.

### 1.4. Mild Hypothermia

Mild hypothermia (Over 28°C) (mH) was not sufficient to get EEG electric silence in 100% of pts, and estimated safe time is only 10 minutes. One study found that in order to achieve cerebral electrical inactivity in >95% of patients it was needed to lower the temperature to 12.7°C [3].

The brilliant results obtained with the aid of selective anterograde cerebral perfusion, led to a progressive reduction of the circulatory arrest temperature, with the rise of safe time along with a reduction of the CPB time, as a consequence of the reduction of time needed to cool and rewarm the patient [4] and of the hypothermia-related side effects.

However, there is still no definitive consensus concerning the optimal range of temperature to be used during circulatory arrest [5%^9^].

**Figure 1:**
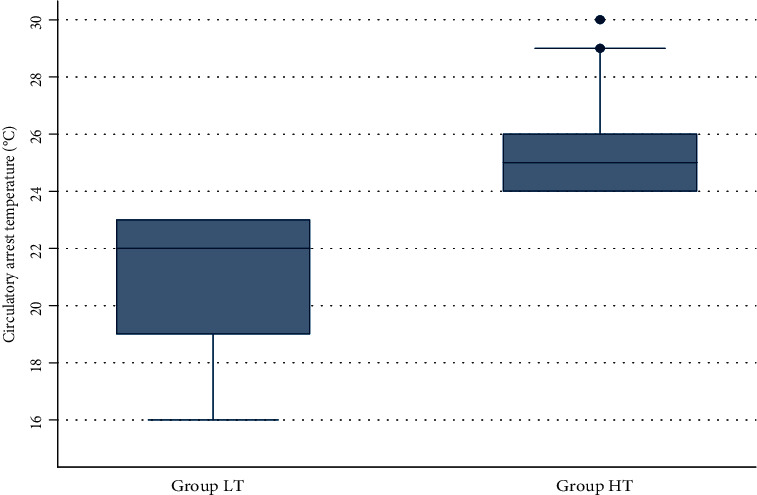
Box plot showing distribution of temperature over our population.

This study examined 16-year trends in the presentation, diagnosis, hospital outcome and treatment of type A AAD in our Cardiac Surgery Unit at Policlinico Umberto I of Rome. We focused our analysis on the differences between two strategies on CPB temperature conduction: lower (*T* < 24°C) versus higher (*T* ≥ 24°C) circulatory arrest temperature (Figure 1). ACP was performed in all patients.

## 2. Methods

### 2.1. Patient Selection

Data were collected on a population of 241 patients who presented with AAD from August 2002 through March 2018 and underwent surgery for type A Aortic dissection at Policlinico Umberto I Cardiac Surgery Department with ACP strategy. Patients were divided into 2 groups reflecting the differences in lowest temperature value on CPB during this 16-years observation.

Data on patient demographic characteristics, presenting history, physical examination, imaging studies, management, and hospital outcomes were collected from our institutional database.

All patients were invasively monitored during surgery with Trans-Esophageal Echography (TEE) and at least one arterial line and a large peripheral venous line. One central venous access was used routinely, almost always represented by a multi-lumen jugular vein catheter. Both bladder and nasopharyngeal temperatures were monitored. Neurologic monitoring was accomplished by (Near Infrared Cerebral Saturation (INVOS™ 5100C Cerebral/Somatic Oximeter, Medtronic, Minneapolis, MN).

Anesthesia protocol was maintained unchanged during the study and consisted of intravenous anesthesia technique based on propofol TCI (1.5 mg/ml) supplemented by continuous infusion of Sufentanil (0.35–0.5 mcg/kg/hr) and refracted boluses of muscle relaxants with BIS™ control of adequate hypnosis level.

The rewarming process had not undergone into any modification over the years. First of all, adequate duration was maintained to allow homogeneous brain warming (about 30 minutes) considering that the brain receives about 15% of cardiac output, with the maintenance of a thermal gradient between the water of the thermo-circulator and venous blood of the circuit below (10°C). For better maintenance of cerebral self-regulation and optimization of cellular enzymatic activity alpha-stat strategy is used. Finally, it must not be forgotten that the temperature rise with respect to the time unit (°C/minutes) is also a function of the pump flow, which in turn is also a function of the surgical needs as well as of the patient's metabolic needs.

We considered as adverse outcomes: perioperative mortality; neurological deficits; focal deficits; acute kidney injury requiring dialysis, intestinal ischemia; need for post-operative tracheostomy and peri-operative infections. “Neurological deficits” were defined as both temporary and permanent significant deficits, discovered during the post-operative period. “Focal deficits” consisted of a set of symptoms or signs in which causation could be localized to an anatomic site in the central nervous system [10]. “Acute kidney injury (AKI) requiring dialysis” was defined as a loss of kidney function or terminal kidney failure according to KIDGO criteria.

“Intestinal ischemia” was defined as a condition related to a reduction of splanchnic hematic flow, that could be caused by heart failure and aggravated by inotrope use and/or vasopressors and by atherosclerotic plaques [11]. “Infections” were suspected when as at least a single white cell value above 12.000 cell/mm^3^ was recorded along with a temperature above 38°C and an increased value of PCT during the post-operative period, criteria believed to have a high specificity for infections [12, 13].

Data were analyzed retrospectively to investigate whether a statistical association existed between the previously considered adverse outcomes and the temperature ranges. Group data were analyzed for historical trends in demographic characteristics, presentation, evaluation, management, and hospital outcomes.

### 2.2. Statistical Analysis

Categorical data were presented as absolute frequencies and percent values. Quantitative measurements were expressed as mean ± SD and were checked for testing the normality of distribution (Shapiro–Wilk test) either in the overall patients as well as in the two groups of patients separately considered. In addition, Levene's test was used for testing the homogeneity of variance between groups. Due to non-normality of data and/or dishomogeneity of variance for some variables, the two groups of patients (Group LT: arrest circulation temperature < 24°C; Group HT: higher arrest temperature ≥24) were compared by Mann-Whitney test for the quantitative variables. The categorical variables were compared by Fisher's exact probability test (in case of two by-two contingency tables) or chi-square test. Bonferroni's correction was applied in case of multiple comparisons to control the experiment wise Type I error probability. H_1_ was postulated bidirectional for all the analyses. A *p* value of <0.05 was considered statistically significant. Statistical analysis was performed using the STATA Statistical Software: Release 10, College Station, TX: StataCorp LP.

## 3. Results

Across the 16-year period, we did not find relevant differences regarding most of patients' demographics and risks factors, apart from a greater incidence of Marfan syndrome in group HT. We witnessed a difference on symptoms at presentation, with a greater incidence of chest pain and pulse deficits in group HT, a lower incidence of syncope at presentation (Table 1).

In fact, in the group LT the temperature reached lower values (21.1°C ± 2.2°C) in respect of the group HT (25.3°C ± 1.3°C).

**Figure 2:**
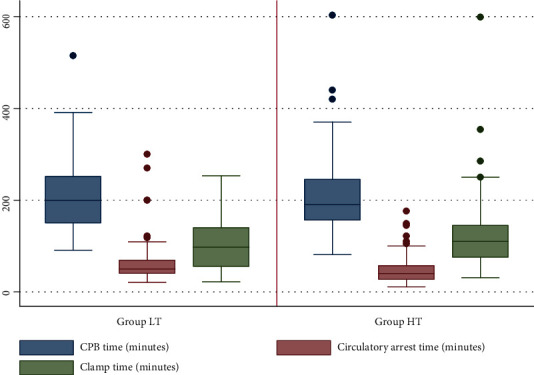
Box plot showing distribution of intraoperatory times over our population. Group LT: Arrest circulation temperature <24°C; Group HT: higher arrest temperature ≥24°C; CPB time: Cardiopulmonary bypass time.

There was no statistical difference between the CPB time over the two groups, but there was a significant difference in the circulatory arrest time, as showed in Table 2 (Figure 2). No significant difference was found regarding the surgical strategy adopted between the two groups (Table 2).

The 30-day mortality rate was 34% in the group LT (32/94) and 21.4% I the group HT (32/147) (*p* = 0.038) (Table 3). In hospital results showed a significant difference between the 2 groups (*p* = 0.019) (Table 3). More specifically, at discharge 58 (61.7%) patients in the group LT and 113 (76.9%) in the group HT were alive. On the other hand, death of all causes occurred in 36 patients in group LT and in 35 patients in group HT. Multiple comparisons demonstrated that despite a similar intraoperative mortality (*p* = NS), a significant greater mortality was observed in the postoperative course in group LT (*p* < 0.03) (Table 3).

We analysed the complications observed during the in-hospital stay, and our results are listed in Table 3.

## 4. Discussion

Acute aortic dissection is a life-threatening condition, and different techniques have been developed to diminish the mortality and the complications of the surgical procedure.

In our analysis, we investigated the optimal temperature range during circulatory arrest dividing our patients in two groups, group LT and group HT: group LT with a deeper hypothermia strategy (*T* < 24°C) and group HT with higher hypothermia strategy (*T* ≥ 24°C). Choosing this temperature as a cut-off for the analysis in this study was fundamentally based on our center experience regarding the temperature to reach during hypothermia that changed over the years, with a progressive approach to higher temperature supported both by the literature and our results [14, 15].

Despite the cardiopulmonary times were not significantly different between the 2 groups, HCA time was significantly longer in the lower temperature (LT) group without any significant difference regarding the surgical strategy performed. Since, no changes occurred in our rewarming protocol, this results may be attributed to the fact that our surgeons have initiated to practice the surgical treatment of type A aortic dissection almost 18 years ago. And therefore, the difference in the HCA time between the 2 groups and the improvement of the results may be explained by the experience and certainly by the learning curve in this field acquired over 16 years.

We found a significant difference in in-hospital mortality, with a significant reduction in Group HT, and a significant reduction of global incidence of complications.

In our study, the incidence of neurologic complications did not differ between the two groups. A possible explanation for this difference may be that we adopted in our procedures the same protection technique (ACP).

Using moderate hypothermia is correlated to a compromise/suppression of visceral organ metabolism. Physiologically, the kidneys are the most sensitive organs to ischemia, followed by the liver and the bowel. In this study, there was no significant difference in the incidence of AKI requiring dialysis, Chronic Kidney Injury. Even though, we found a statistically significant reduction of intestinal ischemia in group HT. In fact, some studies suggest that the pH decreases and causes intestinal mucosal injury when the intestinal oxygen supply decreases to 50–60% [16].

In our study, furthermore, we observed a reduction of infections in the group HT, even if not statistically significant. Many factors may explain these results. First of all, the pump itself is associated with a broad array of adverse physiologic sequelae that predispose cardiac surgery patients to infectious complications. Cardiopulmonary bypass is known to compromise humoral immunologic defenses, reduce phagocytosis, and activate white blood cells, all of which impair the ability to neutralize infectious organisms. The length of a surgical procedure is also generally correlated with the risk of postoperative infection [17]. Secondly, the gastrointestinal bacterial population is an estimated at 1014; when the mucosal barrier is destroyed, these bacteria translocate across the intestines into the vasculature, resulting in severe infection and MODS [18, 19]. In addition, hypoxia disrupts the bacteria themselves, leading to intestinal flora disorder [20].

Finally, it is believed that hypothermia, also, is associated with infections. Possible adverse effects of hypothermia can result from two mechanisms: reduced peripheral blood flow (vasoconstriction) and an impaired immune system. The additional causative factors include higher postoperative protein loss and clotting disturbances, mainly the impaired function of platelets necessary for the initiation of proper healing through platelet plug formation. The above-mentioned disorders may lead to even threefold higher incidence of surgical wound infections and substantially reduced reactivity of the immune system in response to infection.

Since the two groups are similar for type of surgical procedures, we consider these differences depending on the lower temperature reached, according to the current literature. From our analysis, we can consider secure performing aortic surgery in circulatory arrest with a temperature range ≥24°C, with the association of ACP cerebral protection strategy.

## 5. Conclusions

Since we reached a statistical significance on 30-day mortality (*p* = 0.038) and on postoperative death (*p* < 0.03 , see supplementary materials) between those groups, and we did find a global reduction of complications, we can conclude that circulatory arrest performed at temperatures ≥24°C with ACP is a safe strategy for aortic surgery for AAD. In particular, since in our analysis we found that group HT mean temperature was of 25.3°C, we believe that a temperature between 25 and 26°C is safe for circulatory arrest during type A AAD.

## Figures and Tables

**Table 1:** Demographics and preoperative baseline characteristics.

Variables	All pts	Group LT	Group HT	*p* value^∗^
	(*n* = 241)	(*n* = 94)	(*n* = 147)	
	Mean ± SD	Mean ± SD	Mean ± SD	
	*n* (%)	*n* (%)	*n* (%)	
Age, years	63.2 ± 11.7	62.1 ± 10.7	63.9 ± 12.3	0.152
Gender (male)	160 (66.4)	66 (70.2)	94 (64.0)	0.331
*Comorbidities*				
Hypertension	208 (86.3)	79 (84.0)	129 (87.8)	0.446
Diabetes mellitus	24 (10.0)	8 (8.5)	16 (10.9)	0.661
Atherosclerosis	101 (41.9)	33 (35.1)	68 (46.3)	0.108
Marfan syndrome	12 (5.0)	1 (1.1)	11 (7.5)	**0.031**
Previous cardiac surgery	7 (2.9)	2 (2.1)	5 (3.4)	0.708
*Symptoms at admission*				
Shock	40 (16.6)	13 (13.8)	27 (18.4)	0.381
Severe or worst-ever pain	223 (92.5)	82 (87.2)	141 (95.9)	**0.021**
Chest pain	202 (83.8)	67 (71.3)	135 (91.8)	**<0.001**
Syncope	37 (15.4)	22 (23.4)	15 (10.2)	**0.010**
Pulse deficits on presentation	110 (45.6)	33 (35.1)	77 (52.4)	**0.012**

^∗^ *p* values refer to Mann–Whitney test for the quantitative data and to Fisher's exact probability test for the categorical data. pts: patients, Group LT: arrest circulation temperature <24°C, Group HT: higher arrest temperature ≥24°C.

**Table 2:** Intraoperative data.

Variables	All pts	Group LT	Group HT	*p* value^∗^
	(*n* = 241)	(*n* = 94)	(*n* = 147)	
	Mean ± SD	Mean ± SD	Mean ± SD	
	*n* (%)	*n* (%)	*n* (%)	
Cardiopulmonary bypass time (minutes)	208.4 ± 77.1	210.7 ± 78.5	206.9 ± 76.4	0.742
Cross-clamp time (minutes)	111.1 ± 63.1	102.3 ± 53.3	116.8 ± 68.2	0.101
Circulatory arrest time (minutes)	52.3 ± 35.8	62.7 ± 44.7	45.7 ± 26.8	**<0.001**
Circulatory arrest temperature (°C)	23.6 ± 2.7	21.1 ± 2.2	25.3 ± 1.3	**<0.001**
*Surgical strategy*				
(i) Isolate ascending aorta replacement	101 (41.9)	41 (43.6)	60 (40.8)	0.912
(ii) Hemiarch replacement	34 (14.1)	12 (12.8)	22 (15.0)	
(iii) Arch replacement	27 (11.2)	9 ( 9.6)	18 (12.2)	
(iv) Ascending aorta and aortic valve replacement	41 (17.0)	18 (19.1)	23 (15.6)	
(v) Bentall procedure	34 (14.1)	12 (12.8)	22 (15.0)	
(vi) Cabrol procedure	4 (1.7)	2 (2.1)	2 (1.4)	
CABG	32 (13.3)	10 (10.6)	22 (15.0)	0.437

^∗^ *p* values refers to Mann–Whitney test for the quantitative data and to chi square test or Fisher's exact probability test (in case of two-by-two contingency tables) for the categorical data. CABG: Coronary artery bypass graft surgery; pts: patients; Group LT: arrest circulation temperature <24°C; Group HT: higher arrest temperature ≥24°C.

**Table 3:** Results.

Variables	All pts	Group LT	Group HT	*p* value^∗^
	(*n* = 241)	(*n* = 94)	(*n* = 147)	
	Mean ± SD	Mean ± SD	Mean ± SD	
	*n* (%)	*n* (%)	*n* (%)	
Time for intubation (hours)	114.6 ± 171.3	144.0 ± 215.9	96.5 ± 134.5	0.6532
Drainage output over 24 h (ml)	924.1 ± 703.8	1071.3 ± 895.9	833.3 ± 537.4	**0.0353**
Complication (overall)	150 (62.2)	68 (72.3)	82 (55.8)	**0.010**
Limb Ischemia	22 (10.6)	11 (13.9)	11 (8.6)	0.251
Acute kidney injury	65 (31.4)	24 (30.4)	41 (32.0)	0.878
Acute kidney injury requiring dialysis	51 (24.6)	23 (29.1)	28 (21.9)	0.250
Chronic kidney Injury	19 (9.2)	7 (8.9)	12 (9.4)	1.000
Intestinal Ischemia	14 (6.8)	10 (12.7)	4 (3.1)	**0.011**
Neurological deficits	46 (22.2)	20 (25.3)	26 (20.3)	0.491
Focal deficit	38 (18.4)	14 (17.7)	24 (18.8)	1.000
Coma	15 (7.3)	7 (8.9)	8 (6.3)	0.583
Tracheostomy	41 (20.0)	17 (21.8)	24 (18.9)	0.719
Infections	73 (35.3)	34 (43.0)	39 (30.5)	0.074
In-hospital stay (days)	17.3 ± 15.8	19.0 ± 18.9	16.1 ± 13.4	0.3780
30-day mortality	64 (26.6)	32 (34.0)	32 (21.8)	**0.038**
*in-hospital results*				**0.019**
(i) Intraoperative death	34 (14.1)	15 (16.0)	19 (12.9)	
(ii) Postoperative death	36 (14.9)	21 (22.3)	15 (10.2)	
(iii) Alive at discharge	171 (71.0)	58 (61.7)	113 (76.9)	

^∗^ *p* values refers to Mann–Whitney test for the quantitative data and to chi square test or Fisher's exact probability test (in case of two-by-two contingency tables) for the categorical data. pts: Patients; Group LT: arrest circulation temperature <24°C; Group HT: higher arrest temperature ≥24°C.

## Data Availability

Our database used to support the findings of this study are available from the corresponding author upon request. Tables showing the distribution between the 2 groups of the arrest circulation temperature distribution and multiple comparisons regarding the in-hospital results are included within the supplementary information file.

## Additional Points

*Study Limitations.* The main limitation of the current report is that is a retrospective observational study. Our institution switched to the concept of moderate Hypothermia during selective ACP a decade ago, rising the arrest temperature over time, according to surgeon's preferences. In our database we collected data of 10 surgeons, and we did not have a uniform protocol for this procedure due to modifications in our surgical strategies over the years. On the other hand, in our database intraoperative data like utilization of blood products are missing, therefore searching any correlation between these data and complications was not possible.

## Supplementary Materials

Supplementary Materials Tables showing the distribution between the 2 groups of the arrest circulation temperature distribution and multiple comparisons regarding the in-hospital results are included within the supplementary information file.
