# Supplementary material for: Hypothermia during Surgical Treatment of Type A Aortic Dissection: A 16 Years' Experience
Source: Int J Vasc Med. 2020 Jan 25;2020:3893261. doi: 10.1155/2020/3893261 (PMC8339990; doi:10.1155/2020/3893261)
Supplement: Supplementary Materials — Tables showing the distribution between the 2 groups of the arrest circulation temperature distribution and multiple comparisons regarding the in-hospital results are included within the supplementary information file. [file 3893261.f1.docx]

Variable | Obs Mean Std. Dev. Min Max

-------------+--------------------------------------------------------

tempmin | 241 23.63485 2.664479 16 30

**-> grtemp = 0**

tempmin | 94 21.06383 2.208529 16 23

**-> grtemp = 1**

tempmin | 147 25.27891 1.259388 24 30

**Inhospital mortality**

1. **2 groups**

+--------------------+

| Key |

|--------------------|

| frequency |

| expected frequency |

| row percentage |

| column percentage |

| cell percentage |

+--------------------+

| GRTEMP

RISinhosp | 0 1 | Total

-----------+----------------------+----------

0 | 58 113 | 171

| 66.7 104.3 | 171.0

| 33.92 66.08 | 100.00

| 61.70 76.87 | 70.95

| 24.07 46.89 | 70.95

-----------+----------------------+----------

1 | 36 34 | 70

| 27.3 42.7 | 70.0

| 51.43 48.57 | 100.00

| 38.30 23.13 | 29.05

| 14.94 14.11 | 29.05

-----------+----------------------+----------

Total | 94 147 | 241

| 94.0 147.0 | 241.0

| 39.00 61.00 | 100.00

| 100.00 100.00 | 100.00

| 39.00 61.00 | 100.00

**Fisher's exact = 0.014**

1. **3 groups**

| inhospital results   - *intraoperative death* - *postoperative death* - *alive at discharge* | 34 (14.1)  36 (14.9)  171 (80.0) | 15 (16.0)  21 (22.3)  58 (61.7) | 19 (12.9)  15 (10.2)  113 (76.9) | **chi2 = 0.019** |
| --- | --- | --- | --- | --- |

**RISULTATI** | GRTEMP

| 0 1 | Total

-----------+----------------------+----------

Viventi | 58 113 | 171

| 66.7 104.3 | 171.0

| 33.92 66.08 | 100.00

| 61.70 76.87 | 70.95

| 24.07 46.89 | 70.95

-----------+----------------------+----------

Postop. | 21 15 | 36

death | 14.0 22.0 | 36.0

| 58.33 41.67 | 100.00

| 22.34 10.20 | 14.94

| 8.71 6.22 | 14.94

-----------+----------------------+----------

Intraop. | 15 19 | 34

death | 13.3 20.7 | 34.0

| 44.12 55.88 | 100.00

| 15.96 12.93 | 14.11

| 6.22 7.88 | 14.11

-----------+----------------------+----------

Total | 94 147 | 241

| 94.0 147.0 | 241.0

| 39.00 61.00 | 100.00

| 100.00 100.00 | 100.00

| 39.00 61.00 | 100.00

**Pearson chi2(2) = 7.8865 Pr = 0.019**

**Multiple comparisons**

**After the intervention**

| col

row | 1 2 | Total

-----------+----------------------+----------

Viventi | 58 113 | 171

| 33.92 66.08 | 100.00

| 73.42 88.28 | 82.61

-----------+----------------------+----------

Postop. | 21 15 | 36

death | 58.33 41.67 | 100.00

| 26.58 11.72 | 17.39

-----------+----------------------+----------

Total | 79 128 | 207

| 38.16 61.84 | 100.00

| 100.00 100.00 | 100.00

**Fisher's exact: p = 0.008;**

**Fisher's exact test with Bonferroni’s correction: p < 0.03**

**Intraoperative**

| col

row | 1 2 | Total

-----------+----------------------+----------

Viventi | 58 113 | 171

| 33.92 66.08 | 100.00

| 79.45 85.61 | 83.41

-----------+----------------------+----------

Intraop. | 15 19 | 34

death | 44.12 55.88 | 100.00

| 20.55 14.39 | 16.59

-----------+----------------------+----------

Total | 73 132 | 205

| 35.61 64.39 | 100.00

| 100.00 100.00 | 100.00

**Fisher's exact p = 0.327;**

**Fisher's exact test with Bonferroni’s correction: p = NS**
